# CD147-expressed small extracellular vesicles enhance the detection of colorectal neoplasia with fecal immunochemical test

**DOI:** 10.1016/j.esmogo.2025.100140

**Published:** 2025-02-13

**Authors:** L.-C. Chang, B.-R. Lin, H.-M. Chiu, M.-S. Wu, T. Ochiya, T.-L. Shen

**Affiliations:** 1 Department of Internal Medicine, National Taiwan University Hospital, Taipei, Taiwan; 2 Health Management Center, National Taiwan University Hospital, Taipei, Taiwan; 3 Department of Surgery, National Taiwan University Hospital, Taipei, Taiwan; 4 Division of Molecular and Cellular Medicine, Institute of Medical Science, Tokyo Medical University, Tokyo, Japan; 5 Department of Plant Pathology and Microbiology and Center for Biotechnology, National Taiwan University, Taipei, Taiwan; 6 Center of Biotechnology, National Taiwan University, Taipei, Taiwan

**Keywords:** colorectal cancer, CD147, small extracellular vesicle, biomarker, blood test

## Abstract

**Background:**

Blood testing increases adherence to colorectal cancer (CRC) screening. The suboptimal detection of advanced adenoma (AA) and early-stage CRC by blood tests limits their application. The current study aims to investigate the diagnostic performance of CD147-expressed small extracellular vesicles (CD147) in the serum in combination with the fecal immunochemical test (FIT) for detecting AA and CRC.

**Patients and methods:**

The case-control study enrolled 15 healthy controls and 50 CRC patients in the training cohort and 120 healthy controls, 50 non-AA patients, 50 AA patients, and 60 CRC patients in the validation cohort. CD147 was detected using the ExoScreen assay. The sensitivity and specificity of CD147 alone and combined with the FIT for detecting colorectal neoplasia were compared.

**Results:**

CD147 could detect AA and CRC with a sensitivity of 58.0% and 71.7%, respectively. Combined with a 1-day FIT, the sensitivity for detecting AA and CRC would increase to 78.0% and 93.3%, respectively. Moreover, CD147 with 1-day FIT had a higher sensitivity than 1-day FIT alone for detecting proximal CRC (100% versus 89.5%). Besides, CD147 may detect 57.7% and 50.0% of the AA and CRC that FIT failed to detect, respectively. However, the specificity and false-positive rate of CD147 were inferior to those of FIT for detecting AA and CRC.

**Conclusions:**

CD147 is superior to FIT for detecting AA and proximal neoplasia. Moreover, CD147 can increase the sensitivity of FIT for detecting either AA or CRC by combinatory use. However, false positivity is the concern of CD147, which should be further resolved in the future.

## Highlights

• This study highlights the value of CD147⁺ sEVs as biomarkers for detecting colorectal neoplasia.
• It highlights CD147's superiority over FIT in detecting precancerous lesions and proximal neoplasia for early detection.
• Combining CD147 with FIT boosts sensitivity for advanced adenoma and CRC, even detecting FIT-negative cases.
• Unlike current blood tests (e.g., ctDNA tests), CD147+ sEVs outperform in detecting AA and early-stage CRC.
• These findings suggest a promising method to improve CRC screening, though larger prospective validation is needed.

## Introduction

Colorectal cancer (CRC) ranks as the third most common cancer and the second leading cause of cancer-related deaths worldwide.^1^ Early detection through screening could significantly reduce the mortality from CRC.^2^ The implementation of CRC screening has reduced the CRC incidence rate by 3.3% per year from 2011 to 2016.^3^ The most commonly used modalities for screening CRC are fecal immunochemical test (FIT) and colonoscopy. Colonoscopy plays a role in confirmatory diagnosis, and colonoscopy-based screening may reduce the mortality rate by 67%.^4^^,^^5^ However, colonoscopy has limited adherence because of the inconvenience and invasiveness. FIT is the most common modality for screening worldwide. Although FIT effectively reduces CRC mortality, its adherence is suboptimal because of the negative acceptance associated with fecal specimen collection. A blood test could provide an additional 6.5% participation than FIT for screening.^6^ Not surprisingly, there is a preference for blood-based screening to improve the limited acceptance of either colonoscopy or FIT. Moreover, FIT has limitations in detecting advanced adenoma (AA), early-stage CRC, and proximal CRC, with one in five post-FIT CRCs being interval cancers, which reduces the effectiveness of screening programs and participation rates, underscoring the urgent need for more reliable noninvasive tests to accurately detect precancerous, early-stage, and proximal neoplasms and improve screening outcomes.^7^, ^8^, ^9^ Methylated septin 9 (mSEPT9) is a blood test approved by the United States Food and Drug Administration for CRC screening. The sensitivity of mSEPT9 for detecting CRC and AA is 48.2% and 11.2%, respectively.^10^ The suboptimal sensitivity of the currently available blood test for detecting either CRC or precancerous neoplasia highlights the need and potential merit for developing a novel blood-based test for detecting colorectal neoplasia.

Small extracellular vesicles (sEVs) are membrane-bound vesicles ranging from 30 to 150 nm in diameter and encapsulate varied proteins, nucleic acids, and other biomolecules with either the physiological or pathological status of parental cells.^11^ sEVs transport biomolecules between cells and play an essential role in intercellular communication. Moreover, they contribute to cancer formation by facilitating tumor immunity, angiogenesis, metastasis, etc. The sEVs isolated from breast cancer demonstrated stable phosphorylated proteins and provided a window to investigate the cancer progression and metastasis.^12^ Meanwhile, sEVs have been considered helpful for early cancer detection as a biomarker. For example GPC1 (glypican-1)-expressed sEVs may help to diagnose pancreatic cancer^13^ and CRC,^14^ respectively. CD147 modulates matrix metalloproteinase activity and is essential in cancer development.^15^ In the experiment with cell line, CD147-expressed sEVs (CD147) can promote differentiation of CRC stem cells.^16^ Moreover, the CD147 in blood from the CRC patients is also higher than that from the healthy subjects.^17^ Previous study also established ExoScreen, a sensitive and rapid analytical technique, for detecting CRC using CD147 as a biomarker.^18^

This study investigated the performance of CD147 in serum for detecting CRC and AA as a biomarker in combination with FIT. We identified a diagnostic cut-off in the training cohort and further validated it in an independent cohort to clarify the sensitivity of CD147 combined with FIT for detecting colorectal neoplasia.

## Patients and methods

### Patients

Patients who underwent colonoscopy at National Taiwan University Hospital were included prospectively. The blood samples in the training and validation sets were collected chronologically. Participants with a history of inflammatory bowel disease or hereditary CRC, such as Lynch syndrome, familial adenomatous polyposis, or hyperplastic polyposis, were excluded. Participants having active malignancy within 5 years before a diagnosis of CRC were also excluded.

The study protocol was approved by the Institutional Review Board of the National Taiwan University Hospital (No. 201912055RINB). All the participants signed the informed consent before enrollment.

### Definition

Colorectal neoplasia was classified as a non-advanced adenoma (non-AA), AA, and CRC according to the 2019 World Health Organization classification system for tumors of the digestive system.^19^ AA was defined as a lesion ≥10 mm in diameter or with a villous component or high-grade dysplasia. Advanced neoplasia (AN) was defined as AA and CRC. Healthy control was defined as a subject with a negative colonoscopy. Cecum to splenic flexure was defined as the proximal colon.

### Fecal immunochemical test

All study subjects were asked to submit one spot stool sample for 2 consecutive days before the colonoscopy. The collected samples were refrigerated during storage and returned to the hospital on the colonoscopy day. The submitted fecal samples were then sent to the central clinical laboratory for assay on the same day. THE OC-SENSOR FIT kit (Eiken Chemical Co., Ltd., Tokyo, Japan) with 20 μg hemoglobin/g of feces (μg Hb/g) was used to determine FIT positivity. The laboratory technologists were all blinded to the colonoscopy findings. The positivity of 2-day FIT was defined as at least one of the two tests being positive.

### Blood collection and reagents

Whole blood was collected in BD Vacutainer™ SST™ II Advanced (Avantor lnc., Radnor Township, PA) tube and processed within 2 h after blood was drawn. The serum was aliquoted and kept at −80°C until used, and freeze-thawing was avoided. The details of antibodies used for immunoblotting are described in Supplementary Methods, available at https://doi.org/10.1016/j.esmogo.2025.100140.

### ExoScreen assay and enzyme-linked immunosorbent assay

A 96-well half-area white plate was used for ExoScreen and enzyme-linked immunosorbent assay. The details of the experiments are described in Supplementary Methods, available at https://doi.org/10.1016/j.esmogo.2025.100140, and previous study.^18^

### Statistical analysis

The signal density of CD147 was tested by cancer stage, anatomic location of CRC, and tumor size. The tumor size was divided by the quarter method. To obtain the diagnostic discrimination ability of CD147 for distinguishing CRC from healthy control, an investigation with the receiver operating characteristic (ROC) curve was carried out. The optimal cut-off of the signal density of CD147 for diagnosing CRC was estimated by the highest Youden index.^20^ The cut-off obtained from the training cohort was tested in the validation cohort. The area under curve (AUC) of CD147, CEA, and FIT for detecting CRC was compared in the validation cohort. The primary outcome measurements included sensitivity, specificity, true-positive rate (TPR), and false-positive rate (FPR). The diagnostic performance between CD147 and FIT was also compared by relative TPR (rTPR) and relative FPR (rFPR). The rTPR and rFPR were defined as TPR_CD147_/TPR_FIT_ and FPR_CD147_/FPR_FIT_, respectively. The statistical analysis was conducted using SAS 9.4 for Windows (SAS Institute, Inc., NC).

## Results

### Demographic and clinical information

A total of 65 and 280 subjects were enrolled in the training and validation cohorts, respectively. The demographics are listed in Table 1. In the training cohort, the indication for colonoscopy included screening (44.6%), surveillance (9.2%), and symptomatic (46.2%). Fifteen healthy controls, 8 stage I CRC patients, 18 stage II CRC patients, 19 stage III CRC patients, and 5 stage IV CRC patients were enrolled in the training set. Among the CRCs, 27 (41.5%) lesions were in the proximal colon. In the validation cohort, 47.9%, 16.4%, and 35.7% of the subjects received colonoscopy for screening, surveillance, and symptomatic, respectively. A total of 120 healthy controls, 50 non-AA patients, 50 AA patients, 15 stage I CRC patients, 15 stage II CRC patients, 15 stage III CRC patients, and 15 stage IV CRC patients were enrolled for the validation cohort. Among the lesions, 115 (41.1%) were located in the proximal colon.

**Table 1:** Demographics and clinical information of the study subjects

	Training set *n* = 65	Validation set *n* = 280
Mean age, years (SD)	66.5 (12.3)	60.6 (11.9)
Gender		
Male, *n* (%)	36 (55.4)	143 (51.1)
Female, *n* (%)	29 (44.6)	137 (48.9)
Smoking, *n* (%)		
Non-smoker	56 (86.2)	203 (72.5)
Current smoker (all tobacco)	3 (4.6)	33 (11.8)
Ex-smoker	6 (9.2)	44 (15.7)
Indication for colonoscopy, *n* (%)		
Screening	29 (44.6)	134 (47.9)
Surveillance	6 (9.2)	46 (16.4)
Symptomatic	30 (46.2)	100 (35.7)
Pathology, *n* (%)		
No adenoma	15 (23.1)	120 (42.8)
Non-AA	—	50 (17.8)
AA	—	50 (17.8)
Cancer stage		
Stage I	8 (12.3)	15 (5.4)
Stage II	18 (27.7)	15 (5.4)
Stage III	19 (29.2)	15 (5.4)
Stage IV	5 (7.7)	15 (5.4)
Proximal lesion, *n* (%)	27 (41.5)	115 (41.1)

AA, advanced adenoma; SD, standard deviation.

### Establish the signal intensity cut-off of CD147 for detecting CRC

**Figure 1:**
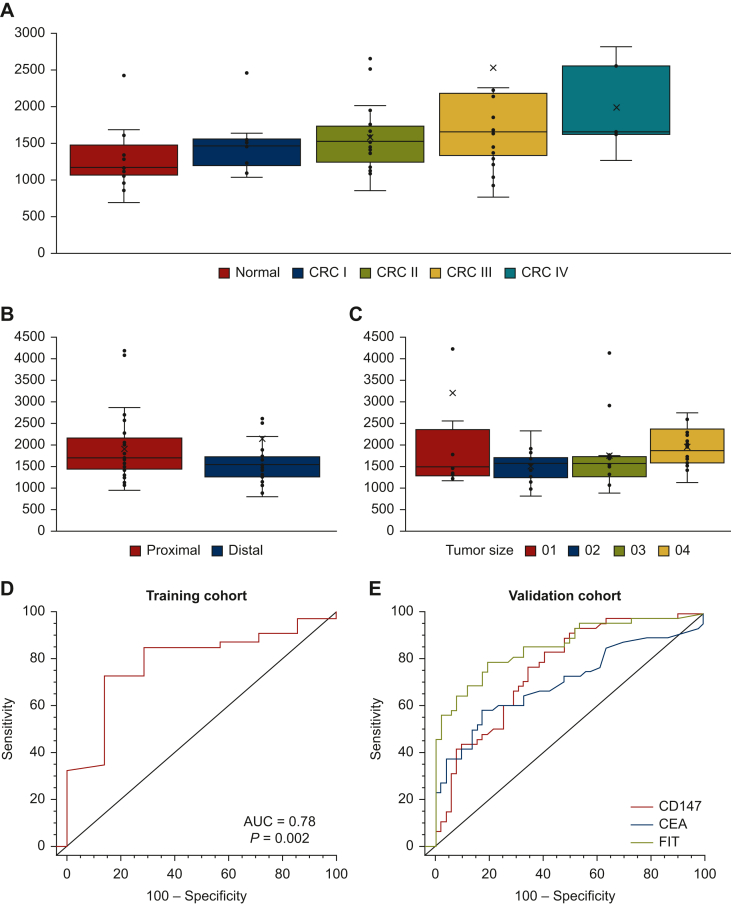
**The signal intensity of CD147 in the serum of subjects with CRC.** The signal intensity of CD147 had a trend toward increasing with the cancer stage, but the increase was not statistically significant (*P* for trend = 0.76) (A). The signal intensity was not significantly different between the proximal and distal colon cancer (*P* = 0.66) (B). The tumor size of CRC was subclassified by the quarter method, and the signal intensity did not increase with the tumor size (*P* = 0.23) (C). The diagnostic performance of CD147 expressed on small extracellular vesicles for detecting CRC was demonstrated by the receiver operating characteristic curve. In the training cohort, CD147 could distinguish CRC from healthy control with the AUC of 0.78 and a *P* value of 0.002 (D). In the validation cohort, the AUC of CD147, FIT, and CEA for detecting CRC was 0.77, 0.86, and 0.70, respectively (E). AUC of FIT was higher than the blood test, including CD147 and CEA. However, the blood test was more convenient than FIT and could increase the screening attendance rate, thereby improving the effectiveness of screening. AUC, area under curve; CEA, carcinoembryonic antigen; CRC, colorectal cancer; FIT, fecal immunochemical test.

The CD147 signal intensity increased with the cancer stage despite the trend being non-significant (Figure 1A, *P* for trend = 0.76). Similarly, the CD147 signal intensity was not significantly different by anatomic location (Figure 1B, *P* = 0.66) and tumor size (Figure 1C, *P* = 0.23) of cancer. The cut-off of the CD147 signal density, which provided the most optimal accuracy for discriminating CRC, was estimated by the highest Youden index.^20^ The ROC curve of CD147 for detecting CRC in the training set is shown in Figure 1D. CD147 could detect CRC with the AUC and *P* value of 0.78 and 0.002, respectively. The cut-off obtained from the training cohort was tested in the validation cohort. The AUC of CD147, CEA, and FIT for detecting CRC was compared in the validation cohort, and their AUCs for detecting CRC were 0.77, 0.70, and 0.86, respectively (Figure 1E).

### Sensitivity and specificity of CD147 in combination with the FIT for detecting colorectal neoplasia

**Figure 2:**
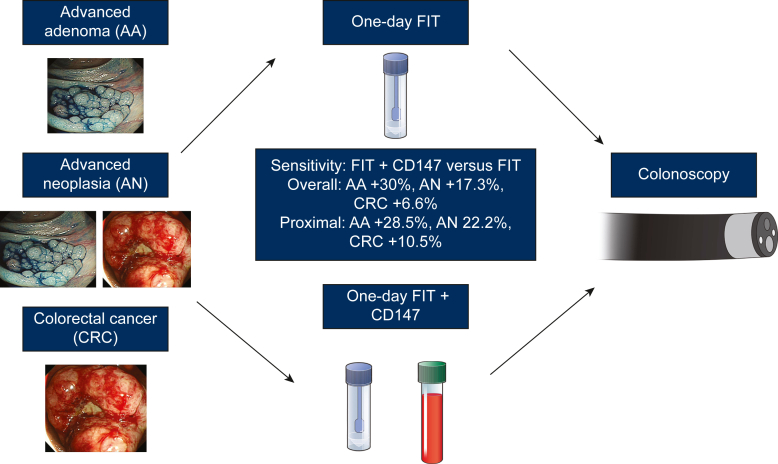
**The comparison of sensitivity for detecting overall and proximal colorectal neoplasia between 1-day FIT and CD147 in combination with 1-day FIT.** For detecting the overall colorectal neoplasia, CD147 in combination with 1-day FIT could increase sensitivity by 30% for AA, 17.3% for AN, and 6.6% for CRC compared with 1-day FIT alone. For detecting proximal colorectal neoplasia, CD147 in combination with 1-day FIT could increase the sensitivity by 28.5% for AA, 22.2% for AN, and 10.5% for CRC compared with 1-day FIT. AA, advanced adenoma; AN, advanced neoplasia; CRC, colorectal cancer; FIT, fecal immunochemical test.

The sensitivity and specificity of CD147 and FIT for detecting colorectal neoplasia are listed in Table 2. For detecting either adenoma or AA, CD147 had a higher sensitivity than 1-day FIT (adenoma: 58.0% versus 45.0%; AA: 58.0% versus 48.0%) or 2-day FIT (adenoma: 58.0% versus 46.0%; AA: 58.0% versus 50.0%). However, CD147 had a lower sensitivity than FIT for detecting CRC, especially in stage II-IV cancer. CD147 may improve FIT’s sensitivity for detecting both AA and CRC. Nevertheless, in combination with CD147, the sensitivity of 1-day FIT for detecting AA and CRC may increase from 48.0% to 78.0% and 86.7% to 93.3%, respectively. The comparison of sensitivity for detecting colorectal neoplasia between 1-day FIT and CD147 plus 1-day FIT is shown in Figure 2. Unexpectedly, the specificity of FIT decreased after combining with CD147. The age, gender, and tumor characteristics of subjects with positive results from 1-day FIT and 1-day FIT plus CD147 are presented in Supplementary Table S1, available at https://doi.org/10.1016/j.esmogo.2025.100140. Compared with subjects with a positive FIT, those with a positive FIT plus CD147 were younger (61.1 versus 62.5 years), were predominantly female (46.7% versus 44.7%), showed a higher detection rate of AA (17.3% versus 16.0%), and had a higher detection rate of proximal lesions (39.1% versus 34.7%). However, the differences were not statistically significant.

**Table 2:** Comparison of sensitivity and specificity between CD147 and FIT in combination

		Adenoma^a^*n* = 100	AA *n* = 50	AN *n* = 110	CRC *n* = 60	CRC-I *n* = 15	CRC-II *n* = 15	CRC-III *n* = 15	CRC-IV *n* = 15
1-day FIT	Sensitivity	45.0%	48.0%	69.1%	86.7%	80.0%	93.3%	86.7%	86.7%
	Specificity	41.7%	45.2%	56.5%	55.5%	47.9%	48.7%	48.3%	48.3%
2-day FIT	Sensitivity	46.0%	50.0%	73.6%	93.3%	93.3%	100.0%	93.3%	86.7%
	Specificity	38.3%	42.6%	55.3%	54.1%	46.0%	46.4%	46.0%	45.7%
CD147	Sensitivity	58.0%	58.0%	65.4%	71.7%	80.0%	66.7%	73.3%	66.7%
	Specificity	39.4%	39.6%	43.5%	43.2%	41.1%	40.4%	40.8%	40.4%
CD147 + 1-day FIT	Sensitivit Specificity	80.0%	78.0%	86.4%	93.3%	93.3%	93.3%	93.3%	93.3%
		19.4%	19.1%	23.5%	23.2%	20.4%	20.4%	20.4%	20.4%
CD147 + 2-day FIT	Sensitivity	81.0%	80.0%	89.1%	96.7%	93.3%	100.0%	100.0%	93.3%
	Specificity	17.2%	17.4%	22.4%	21.8%	18.5%	18.9%	18.9%	18.5%

AA, advanced adenoma; AN, advanced neoplasia; CRC, colorectal cancer; CRC-I/II/III/IV, stage I/II/III/IV CRC; FIT, fecal immunochemical test.

a Adenoma included non-AA and AA.

### Sensitivity and specificity of CD147 for detecting proximal colorectal neoplasia

CD147 had a higher sensitivity than 1-day FIT for detecting proximal AA (51.4% versus 42.9%) and AN (61.1% versus 59.3%), respectively. The sensitivity for detecting proximal advanced lesions may improve if the FIT is combined with CD147. In combination with CD147, the sensitivity of FIT for detecting proximal AA and AN could improve from 42.9% to 71.4% and 59.3% to 81.5%, respectively. In contrast, in combination with CD147, FIT may detect 100% proximal colon cancer across stage I-IV, with a specificity of 28.1% (Table 3). The comparison of sensitivity for detecting proximal colorectal neoplasia between 1-day FIT and CD147 plus 1-day FIT is shown in Figure 2.

**Table 3:** Comparison of sensitivity and specificity between CD147 and FIT for detecting proximal neoplasm

		Proximal colon						
		AA *n* = 35	AN *n* = 54	CRC *n* = 19	CRC-I *n* = 5	CRC-II *n* = 5	CRC-III *n* = 4	CRC-IV *n* = 5
1-day FIT	Sensitivity	42.9%	59.3%	89.5%	100.0%	100.0%	75.0%	80.0%
	Specificity	53.8%	67.2%	63.5%	57.3%	57.3%	55.9%	56.4%
2-day FIT	Sensitivity	45.7%	61.1%	89.5%	100.0%	100.0%	75.0%	80.0%
	Specificity	52.5%	65.6%	61.5%	55.5%	55.5%	54.1%	54.6%
CD147	Sensitivity	51.4%	61.1%	79.0%	100.0%	80.0%	75.0%	60.0%
	Specificity	38.8%	44.3%	45.8%	43.6%	42.7%	42.3%	41.8%
CD147 + 1-day FIT	Sensitivity	71.4%	81.5%	100.0%	100.0%	100.0%	100.0%	100.0%
	Specificity	21.3%	27.9%	28.1%	24.6%	24.6%	24.3%	24.6%
CD147 + 2-day FIT	Sensitivity	74.3%	83.3%	100.0%	100.0%	100.0%	100.0%	100.0%
	Specificity	20.0%	26.2%	26.0%	22.7%	22.7%	22.5%	22.7%

AA, advanced adenoma; AN, advanced neoplasia; CRC, colorectal cancer; CRC-I/II/III/IV, stage I/II/III/IV CRC; FIT, fecal immunochemical test.

### Comparison of TPR and FPR between CD147 and FIT

The FIT was the fully qualified test for screening CRC, and we, therefore, compared CD147 with FIT by rTPR and rFPR (Supplementary Figure S1, available at https://doi.org/10.1016/j.esmogo.2025.100140). Compared with the 1-day FIT, CD147 had a better rTPR for detecting AA but worse for detecting AN and CRC. A combination of CD147 and 1-day FIT had a better rTPR for detecting AA, AN, and CRC than 1-day FIT. CD147, combined with the 1-day FIT, had an even better rTPR than the 2-day FIT alone for detecting AA and AN. Regarding rFPR, CD147 was higher than 1-day FIT for detecting AA, AN, and CRC. The rFPR became higher if CD147 was combined with the FIT.

### Detection of CD147 for detecting FIT-negative colorectal neoplasia

The sensitivity of CD147 for detecting FIT-negative colorectal neoplasia is demonstrated in Table 4. There were 8 FIT-negative CRCs, 34 FIT-negative ANs, and 26 FIT-negative AAs in the validation cohort for 1-day FIT. In combination with FIT, CD147 could detect an additional 4 (50%) CRCs, 19 (56%) ANs, and 15 (58%) AAs.

**Table 4:** CD147 for detecting FIT-negative colorectal neoplasm

	1-day FIT positive	1-day FIT negative
Advanced adenoma		
CD147 positive, *n* (%)	14 (58.3)	15 (57.7)
CD147 negative, *n* (%)	10 (41.7)	11 (42.3)
Advanced neoplasia		
CD147 positive, *n* (%)	53 (69.7)	19 (55.9)
CD147 negative, *n* (%)	23 (30.3)	15 (44.1)
CRC		
CD147 positive, *n* (%)	39 (75)	4 (50)
CD147 negative, *n* (%)	13 (25)	4 (50)

CRC, colorectal cancer; FIT, fecal immunochemical test.

The clinical information on AN detected by FIT, missed by FIT but detected by CD147, and missed by both FIT and CD147 is summarized in Supplementary Table S2, available at https://doi.org/10.1016/j.esmogo.2025.100140. A total of 15 AAs, 2 stage I CRCs, 1 stage III CRC, and 1 stage IV CRC were missed by FIT but detected by CD147, with 63.2% of these cases located proximally. Compared with cases detected by FIT, these patients were younger (62.8 versus 62.4 years) and predominantly female (52.6% versus 40.8%). Additionally, 11 AAs and 4 CRCs were missed by both FIT and CD147, with 66.7% located proximally. Patients in this group were younger (59.6 years) and predominantly female (66.7%) compared with the two previously mentioned groups.

### Sensitivity and specificity of CD147 combining 1-day FIT with different cut-offs for detecting colorectal neoplasia

FIT detected AA with a sensitivity of 48.0%, 52.0%, and 58.0% at a 20-, 15-, and 10-μg Hb/g cut-off, respectively. The sensitivity of CD147 for detecting AA was 58.0%, which was as sensitive as FIT at the cut-off of 10 μg Hb/g. The sensitivity of CD147 for detecting CRC was 71.7%, which was lower than FIT at three cut-offs. CD147, in combination with FIT at the cut-off of 20 μg Hb/g, had a sensitivity of 93.3% and 78.0% for detecting CRC and AA, respectively. The sensitivity of FIT at the cut-off of 10 μg Hb/g for detecting CRC and AA was 93.3% and 58.0%, respectively. Combination use of CD147 and FIT at the cut-off of 20 μg Hb/g was as sensitive as FIT at the cut-off of 10 μg Hb/g for detecting CRC (93.3% versus 93.3%), but the former had a higher sensitivity than the latter for detecting AA (78.0% versus 58.0%). CD147 combined with FIT at the cut-off of 10 μg Hb/g had a sensitivity of 96.7% for detecting CRC, and its sensitivity for stage I-IV CRC was 100%, 93.3%, 100%, and 93.3%, respectively (Supplementary Table S3, available at https://doi.org/10.1016/j.esmogo.2025.100140).

## Discussion

The present study investigated the diagnostic performance of CD147 protein expressed on the surface of sEVs in serum for detecting colorectal neoplasia. CD147 could detect AA and CRC with a sensitivity of 58.0% and 71.7%, respectively. Combined with the 1-day FIT, the sensitivity for detecting AA and CRC would increase to 78.0% and 93.3%, respectively. Moreover, CD147 had a higher sensitivity than 1-day FIT for detecting proximal AA (51.4% versus 42.9%). Besides, CD147 may detect 57.7% and 50.0% of the FIT-negative AA and CRC, respectively. In summary, combining CD147 with FIT could enhance the sensitivity for detecting significant precancerous neoplasia as well as proximal lesions. Moreover, CD147 may help to detect colorectal neoplasia, which failed to be detected by FIT.

CD147 facilitates the secretion of matrix metalloproteinases from tumor cells, fibroblasts, and endometrial cells. This process destroys the extracellular matrix and basement membrane, significantly promoting tumor cell proliferation, invasion, and metastasis,^15^ while suppressing cell apoptosis and anoikis.^21^ CD147 affects the efficacy of chemotherapy, contributes to neovascularization, and promotes radiation resistance.^22^^,^^23^ CD147 is closely associated with various human malignancies, including liver, lung, breast, colorectal, prostate, and bladder cancers, glioma, and salivary duct carcinoma.^17^^,^^24^, ^25^, ^26^, ^27^ Given its significant role in tumor progression and therapeutic resistance, CD147 is a crucial tumor-related biomarker for disease diagnosis, prognostic assessment, and targeted therapy.^28^

FIT-negative colorectal neoplasia is responsible for FIT interval cancer and is a critical challenge of FIT-based screening. The present study found that CD147 could detect 57.7% and 50.0% of FIT-negative AA and CRC. The result implies that CD147 may help to compensate for the drawback of FIT, thereby providing an opportunity to reduce FIT interval cancer. The sensitivity of FIT for detecting AA and CRC is not perfect and ranges from 23.8% to 27.1% and 65.8% to 73.8%, respectively.^29^^,^^30^ Combining FIT with another FIT or a blood test is a potential resolution to reduce the false negativity of 1-day FIT. CD147 with 1-day FIT had a higher sensitivity than 2-day FIT for detecting AA (78.0% versus 50.0%) and AN (86.4% versus 73.6%). Accordingly, FIT, in combination with CD147, may help to reduce the false negativity of 1-day FIT.

FIT interval cancer accounts for a notable proportion of cases and is associated with right-sided tumors, female sex, and *BRAF* mutations, indicating distinct biological characteristics.^31^ Adding CD147 testing could enhance screening effectiveness but poses cost-effectiveness challenges. One approach is to offer CD147 testing to individuals with repeated FIT-negative results, reducing false negatives but significantly increasing costs, as most will not have neoplasms. Another is to test those nearing the end of screening age to ensure no undetected neoplasms, though this may be less cost-effective with minimal life-year benefits. Further research is needed to assess the feasibility and efficiency of these strategies.

AA and stage I CRC are almost curable diseases after complete resection. Early detection of these lesions is essential for better treatment response. The blood test with mSEPT9 detected AA and stage I CRC with a sensitivity of 11.2% and 35.0%, respectively.^10^ Another blood test using methylated BCAT1/IKZF1 detected AA and stage I CRC with a sensitivity of 9.4% and 41.2%, respectively.^32^ The positivity of circulating tumor DNA is related to the degree of cancer invasiveness (pT stage),^33^^,^^34^ and this causes its suboptimal detection for early lesions. CD147 detected AA and stage I CRC with a better sensitivity than the abovementioned blood tests. Tumor-associated sEV proteins can be detected before the development of a tumor or distal metastasis and suggested sEV proteins as biomarkers for detecting early cancer.^35^

FIT is less sensitive for detecting proximal AN than distal lesions.^36^ FIT sensitivity is lower for detecting sessile serrated lesions and non-polypoid neoplasia, and these lesions are predominantly located in the proximal colon than in the distal part.^37^ Moreover, the magnitude of the hemoglobin degradation is more severe in the proximal neoplasia than in the distal one. The signal intensity of CD147 was not significantly different between the neoplasia located in the proximal and distal colon (Figure 1B, *P* = 0.66). Thus, CD147 helps compensate for FIT’s drawback in detecting proximal lesions.

The false positivity of CD147 is a concern. CD147 increases in CRC and other cancers, including lung cancer, renal cancer, glioblastoma, and hepatoma.^38^, ^39^, ^40^, ^41^ Although the positivity of CD147 in other cancers was lower than in CRC, the FPR may develop. CD147 is also associated with infection and inflammation. The CD147-spike protein may facilitate the infection of coronavirus disease 2019.^42^ CD147 also enhanced human immunodeficiency virus type 1 replication through the cytoplasmic domain.^43^ Moreover, increased CD147 has been implicated in asthma, rheumatoid arthritis, multiple sclerosis, myocardial infarction, and ischemic stroke.^44^ CD147 may increase in the extra-colonic malignancy, infection, or inflammation, contributing to false positivity for detecting colorectal neoplasia.

For optimizing the specificity of CD147, we evaluated the performance of one-step and two-step strategies for detecting CRC using CD147 and the FIT. The one-step strategy defines a positive test as either CD147 or FIT positive, whereas the two-step strategy involves conducting an FIT for subjects with a positive CD147, and only those positive for both CD147 and FIT are provided with a colonoscopy. While the one-step strategy maximizes CRC coverage, it leads to suboptimal specificity. Conversely, the two-step strategy increased the specificity of CD147 for detecting CRC from 43.2% to 54.6%. Furthermore, the sensitivity also increased from 71.7% to 95.4%. However, the two-step strategy missed 2 of 43 (4.7%) CRCs and 15 of 29 (51.7%) AAs. Although the two-step strategy improves both sensitivity and specificity, it risks missing a significant proportion of CRCs and AAs. Therefore, the decision between a one-step and two-step strategy should be made carefully, considering the local CRC prevalence, available resources, workforce constraint, and colonoscopy capacity.

The present study’s first strength is the comprehensive clinical information. We tested CD147, CEA, and twice FIT for every patient before the colonoscopy, which directly helped to compare the diagnostic performance. Another strength is that using protein expressed on sEVs as a biomarker in combination with FIT for detecting CRC is novel and less addressed. Moreover, the result from the training cohort was validated in an independent cohort, making the findings reliable and solid. The validation cohort has only 15 cases in each cancer stage. Therefore, one of the limitations is that the limited case number makes the results of subgroup analysis unstable. Another limitation is that we do not further distinguish the sessile serrated lesion from the conventional adenoma, and the detection rate of CD147 for lesions arising from the serrated pathway cannot be explored.

In conclusion, the present study explored CD147 in serum, effectively detecting colorectal neoplasia. CD147 is superior to FIT for detecting precancerous and proximal neoplasia. CD147 can improve the sensitivity of FIT for detecting either AA or CRC by combination. In contrast to other blood tests by checking circulating tumor DNA tests, CD147 has an advantage for detecting AA and stage I CRC. However, false positivity is the concern of CD147, which should be further resolved in the future. Finally, the diagnostic performance of CD147 in combination with FIT for detecting colorectal neoplasia needs to be further prospectively validated in a larger cohort.
