# Supplementary material for: CD147-expressed small extracellular vesicles enhance the detection of colorectal neoplasia with fecal immunochemical test
Source: ESMO Gastrointest Oncol. 2025 Feb 13;7:100140. doi: 10.1016/j.esmogo.2025.100140 (PMC12836726; doi:10.1016/j.esmogo.2025.100140)
Supplement: Supplementary Material [file mmc1.docx]

**SUPPLEMENTARY FILE**

This supplementary file has been provided by the authors to give readers additional information about their work.

Supplement to: Chang LC *et al*. **“*CD147-expressed Small Extracellular Vesicles Enhance the Detection of Colorectal Neoplasia with Fecal immunochemical Test*”**

**Methods:**

***Blood collection and reagents***

Whole blood was collected in BD Vacutainer™ SST™ II Advanced tubes and processed within two hours of collection. The serum was aliquoted and stored at -80°C, with freeze-thaw cycles minimized as much as possible. The antibodies used for immunoblotting included: mouse monoclonal anti-human CD63 (clone H5C6, 1:200 dilution) from BD Biosciences, mouse monoclonal anti-human CD9 (clone ALB 6, 1:200 dilution) from Santa Cruz Biotechnology, mouse monoclonal anti-human CD147 (clone MEM-M6/1, 1:1,000 dilution) from Novus Biologicals, and mouse monoclonal anti-Actin (clone C4, 1:1,000 dilution) from Millipore. The secondary antibody, horseradish peroxidase-labeled sheep anti-mouse, was obtained from GE HealthCare.

Antibodies for ExoScreen and ELISA were developed by Shionogi & Co., LTD., including mouse monoclonal anti-human CD63 (clone 8A12) and mouse monoclonal anti-human CD9 (clone 12A12). Mouse monoclonal anti-human CD147 (clone MEM-M6/1) was purchased from Novus Biologicals. These antibodies were used to modify either acceptor beads or biotin, following the manufacturer’s protocol. AlphaLISA reagents from Perkin Elmer, Inc. included AlphaScreen Streptavidin-coated donor beads (6760002), AlphaLISA Unconjugated-acceptor beads (6062011), and AlphaLISA Universal buffer (AL001F). AlphaLISA assays were performed in 96-well half-area white plates (6005560) and read using the EnSpire Alpha 2300 Multilabel Plate Reader (Perkin Elmer, Inc.).

***ExoScreen assay***

A 96-well half-area white plate was loaded with 5 µl of the sample, 5 nM biotinylated antibodies, and 50 µg/ml AlphaLISA acceptor beads conjugated to antibodies in universal buffer, with each reagent volume set to 10 µl. The plate was incubated at room temperature for 1 to 3 hours. Without washing, 25 µl of 80 µg/ml AlphaScreen streptavidin-coated donor beads were added, and the reaction mixture was incubated in the dark for another 30 minutes at room temperature. The plate was then read using the EnSpire Alpha 2300 Multilabel Plate Reader, with excitation at 680 nm and emission detected at 615 nm. Background signals from PBS were subtracted from the measured data.

***ELISA***

Ninety-six well plates (Nunc) were coated with 2.5 µg/ml of anti-human CD9 or CD63 antibodies in 50 µl of carbonate buffer (pH 9.6) per well and incubated for 4 hours at room temperature. After two washes with 0.01% Tween-20 in PBS, 100 µl of Blocking One solution (Nacalai Tesque) was added to each well and incubated at room temperature for 1 hour. Following three washes with PBS, serum-derived sEVs were added in a final volume of 50 µl per well and incubated for 1 hour at room temperature.

After three additional PBS washes, 50 µl of biotinylated anti-human CD9 or CD63 antibody, diluted to 1 µg/ml, was added and incubated for 1 hour at room temperature. Following three more washes with PBS, 100 µl of HRP-conjugated streptavidin (Cell Signaling Technology) diluted 1:2,000 in Blocking One solution was added and incubated for 1 hour at room temperature. After a final three washes with PBS, the reaction was developed using Peroxidase (TMB One Component HRP Microwell Substrate, SurModics). The reaction was stopped with Stop Reagent for TMB Microwell Substrates (SurModics), and optical densities were measured at 450 nm.

**Supplementary Table 1.** Demographic and clinical information of subjects with a positive result in one-day FIT and one-day FIT plus CD147

|  | FIT  N=150 | FIT + CD147  N=225 | P value |
| --- | --- | --- | --- |
| Mean age, years (SD) | 62.5 (10.0) | 61.1 (11.3) | 0.20 |
| Gender |  |  |  |
| Male, n (%) | 83 (55.3) | 120 (53.3) | 0.70 |
| Female, n (%) | 67 (44.7) | 105 (46.7) |  |
| Smoking, n (%) |  |  | 0.90 |
| Non-smoker | 102 (68.0) | 158 (70.2) |  |
| Current smoker | 22 (14.7) | 30 (13.3) |  |
| Ex-smoker | 26 (17.3) | 37 (16.4) |  |
| Pathology, n (%) |  |  | 0.21 |
| No adenomas | 53 (35.3) | 89 (39.6) |  |
| Non-AA | 21 (14.0) | 41 (18.2) |  |
| AA | 24 (16.0) | 39 (17.3) |  |
| Cancer stage |  |  |  |
| Stage I | 12 (8.0) | 14 (6.2) |  |
| Stage II | 14 (9.3) | 14 (6.2) |  |
| Stage III | 13 (8.7) | 14 (6.2) |  |
| Stage IV | 13 (8.7) | 14 (6.2) |  |
| Proximal lesion, n (%) | 52 (34.7) | 88 (39.1) | 0.38 |

FIT: fecal immunochemical test, SD: standard deviation, AA: advanced adenoma

**Supplementary Table 2.** Comparison of advanced neoplasia detected by one-day FIT and CD147

|  | FIT+ve  advanced neoplasia  N=76 | FIT-ve/CD147+ve advanced neoplasia  N=19 | FIT-ve/CD147-ve advanced neoplasia  N=15 |
| --- | --- | --- | --- |
| Mean age, years (SD) | 62.4 (11.2) | 62.8 (13.3) | 59.6 (11.8) |
| Gender |  |  |  |
| Male, n (%) | 45 (59.2) | 9 (47.4) | 5 (33.3) |
| Female, n (%) | 31 (40.8) | 10 (52.6) | 10 (66.7) |
| Smoking, n (%) |  |  |  |
| Non-smoker | 48 (63.2) | 16 (84.2) | 13 (86.7) |
| Current smoker | 15 (19.7) | 2 (10.5) | 1 (6.7) |
| Ex-smoker | 13 (17.1) | 1 (5.3) | 1 (6.7) |
| Pathology, n (%) |  |  |  |
| AA | 24 (31.6) | 15 (79.0) | 11 (73.3) |
| Cancer stage, n (%) |  |  |  |
| Stage I | 12 (15.8) | 2 (10.5) | 1 (6.7) |
| Stage II | 14 (18.4) | 0 (0.0) | 1 (6.7) |
| Stage III | 13 (17.1) | 1 (5.3) | 1 (6.7) |
| Stage IV | 13 (17.1) | 1 (5.3) | 1 (6.7) |
| Proximal lesion, n (%) | 32 (42.1) | 12 (63.2) | 10 (66.7) |

advanced neoplasia: colorectal cancer and advanced adenoma, FIT: fecal immunochemical test, SD: standard deviation, AA: advanced adenoma, FIT+ve advanced neoplasia: advanced neoplasia detected by FIT, FIT-ve/CD147+ve advanced neoplasia: advanced neoplasia undetected by FIT but detected by CD147, FIT-ve/CD147-ve advanced neoplasia: advanced neoplasia undetected by neither FIT nor CD147

**Supplementary Table 3.** The sensitivity and specificity of CD147 combining one-day FIT with different cutoffs for detecting colorectal neoplasia

|  |  | Adenoma*  N=100 | AA  N=50 | AN  N=110 | CRC  N=60 | CRC-I  N=15 | CRC-II  N=15 | CRC-III  N=15 | CRC-IV  N=15 |
| --- | --- | --- | --- | --- | --- | --- | --- | --- | --- |
| FIT (10) | Sensitivity  Specificity | 51.0%  35.0% | 58.0%  39.6% | 77.3%  51.2% | 93.3%  49.1% | 93.3%  41.9% | 93.3%  41.9% | 93.3%  41.9% | 93.3%  41.9% |
| FIT (15) | Sensitivity  Specificity | 47.0%  40.0% | 52.0%  43.9% | 70.9%  54.7% | 86.7%  53.2% | 80.0%  46.0% | 93.3%  46.8% | 86.7%  46.4% | 86.7%  46.4% |
| FIT (20) | Sensitivity  Specificity | 45.0%  41.7% | 48.0%  45.2% | 69.1%  56.5% | 86.7%  55.5% | 80.0%  47.9% | 93.3%  48.7% | 86.7%  48.3% | 86.7%  48.3% |
| CD147 | Sensitivity  Specificity | 58.0%  39.4% | 58.0%  39.6% | 65.4%  43.5% | 71.7%  43.2% | 80.0%  41.1% | 66.7%  40.4% | 73.3%  40.8% | 66.7%  40.4% |
| CD147 + FIT (10) | Sensitivity  Specificity | 84.0%  15.6% | 84.0%  15.7% | 90.9%  20.0% | 96.7%  19.1% | 100.0%  16.6% | 93.3%  16.2% | 100.0%  16.6% | 93.3%  16.2% |
| CD147 + FIT (15) | Sensitivity  Specificity | 80.0%  17.8% | 78.0%  17.8% | 86.4%  21.8% | 93.3%  21.8% | 93.3%  19.3% | 93.3%  19.3% | 93.3%  19.3% | 93.3%  19.3% |
| CD147 + FIT (20) | Sensitivity  Specificity | 80.0%  19.4% | 78.0%  19.1% | 86.4%  23.5% | 93.3%  23.2% | 93.3%  20.4% | 93.3%  20.4% | 93.3%  20.4% | 93.3%  20.4% |

*Adenoma included non-advanced adenoma (non-AA) and advanced adenoma (AA).

FIT: fecal immunochemical test, AA: advanced adenoma, AN: advanced neoplasia, CRC: colorectal cancer, CRC-I/II/III/IV: stage I/II/III/IV CRC

**Supplementary Figure 1.** The comparison of true positive rate and false positive rate among CD147, one-day FIT, and two-day FIT


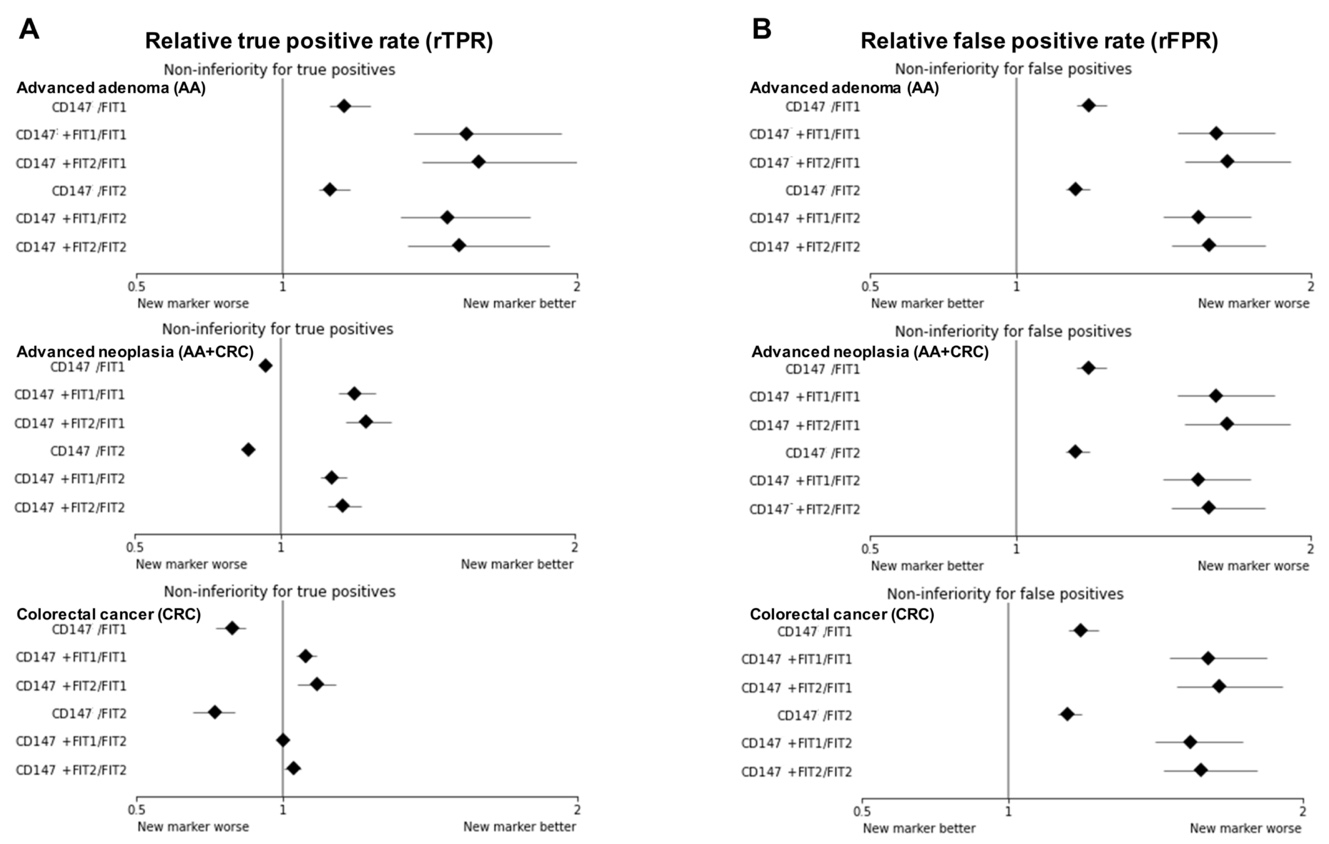


**Figure legend**

The true positive rate was compared to the relative true positive rate. CD147 was superior to either one-day or two-day FIT for detecting advanced adenoma (AA). CD147, in combination with the FIT, was favored for detecting AA than FIT alone. For detecting advanced neoplasia, CD147 was inferior to one-day or two-day FIT. However, CD147, in combination with the FIT, was superior to FIT alone. For detecting CRC, CD147 was inferior to one-day or two-day FIT. CD147 combining one-day or two-day FIT was superior to one-day FIT (**A**). Similarly, the false positivity rate was compared to the relative false positive rate. CD147 was inferior to one-day and two-day FIT. The false positivity increased when CD147 was in combination with the FIT (**B**). (FIT1: one-day FIT; FIT2: two-day FIT).
